# Tailoring Polymer Coatings and Grafting Structures for Photoswitchable Ionic Transport in Solid‐State Nanochannels

**DOI:** 10.1002/asia.202401684

**Published:** 2025-03-12

**Authors:** Yi‐Fan Chen, Vaishali Pruthi, Yu‐Chun Liu, Cheng‐Yeh Yang, Lin‐Ruei Lee, Ming‐Hsuan Chang, Chun‐Chi Chang, Patrick Théato, Jiun‐Tai Chen

**Affiliations:** ^1^ Department of Applied Chemistry National Yang Ming Chiao Tung University 300093 Hsinchu Taiwan; ^2^ Institute for Chemical Technology and Polymer Chemistry (ITCP) Karlsruhe Institute of Technology (KIT) Kaiserstraße 12 D-76131 Karlsruhe Germany; ^3^ Soft Matter Synthesis Laboratory Institute for Biological Interfaces III Karlsruhe Institute of Technology (KIT) Hermann-von-Helmholtz-Platz 1 D-76344 Eggenstein-Leopoldshafen Germany; ^4^ Center for Emergent Functional Matter Science National Yang Ming Chiao Tung University 300093 Hsinchu Taiwan

**Keywords:** Anodic aluminum oxide, Ion conductivity, Photochromism, Photoresponsive, Spiropyran

## Abstract

Photoresponsive ion nanochannels have gained significant attention for their ability to regulate ionic transport in response to external stimuli. The potential of molecular and polymeric architectures in the nanochannels to further enhance and modulate these behaviors, however, remains underexplored. In this work, we explore the integration of spiropyran‐based polymers into anodic aluminum oxide (AAO) nanochannels, resulting in tailored photoresponsive behaviors. Spiropyran undergoes reversible ring‐opening isomerization upon UV irradiation, which leads to changes in the packing and polarity of polymer chains within the nanochannels. The polySp‐coated and polySp‐grafted AAO systems, fabricated via solution wetting and surface‐initiated atom transfer radical polymerization (SI‐ATRP), exhibit unique macroscopic and microscopic responses, including reversible color changes, wettability adjustments, and modulation of ion transport under UV and visible light. These findings demonstrate the potential of spiropyran‐functionalized nanochannels for applications in optical information storage, photogated materials, and sensors. By manipulating molecular architecture and nanoconfinement, this work paves the way for the design of next‐generation photoswitchable systems with enhanced multifunctionality.

## Graphical abstract

Spiropyran‐based polymers have been integrated into anodic aluminum oxide (AAO) nanochannels, resulting in tailored photoresponsive behaviors. The polySp‐coated and polySp‐grafted AAO systems, fabricated via solution wetting and surface‐initiated atom transfer radical polymerization (SI‐ATRP), exhibit unique macroscopic and microscopic responses, including reversible color changes, wettability adjustments, and modulation of ion transport under UV and visible light.

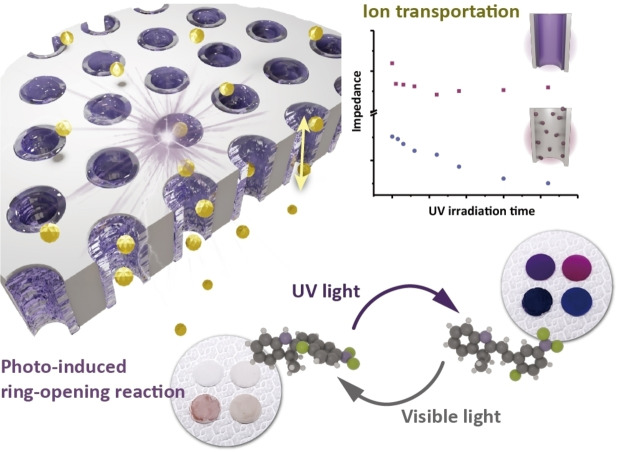

## Introduction

Stimuli‐responsive nanochannels have emerged as versatile platforms for regulating ionic transport,^[1]^ enabling dynamic functionality through external stimuli such as pH,[^2^, ^3^] temperature,[^4^, ^5^] and light.[^6^, ^7^] Among these, photoresponsive nanochannels are particularly promising for advanced applications, including photogate sensors,^[8]^ optical information storage,^[9]^ and light‐controlled ion transport.[^10^, ^11^, ^12^] Light‐responsive materials, such as azobenzene,[^13^, ^14^] nitrobenzyl ester,[^15^, ^16^] coumarin,[^17^, ^18^] and spiropyran,[^19^, ^20^] offer significant advantages, including remote control, high sensitivity, tunable responses, and reversibility.[^6^, ^21^] These materials can trigger photoisomerization, bond formation or cleavage, and polarity inversion, thereby imparting diverse functions to the nanochannels. The tunable confined environments within these nanochannels present exciting opportunities for designing multifunctional systems, where the interplay between molecular structure and nanoconfinement can significantly influence overall behavior.

Various molecular and polymeric architectures have been explored to enhance the functionality of photoresponsive nanochannels.[^22^, ^23^] One common approach is to integrate light‐sensitive molecules, which undergo reversible photoisomerization under light irradiation, into nanochannels via simple modifications, such as nonbonding or bonding to the sidewalls of the channels.[^24^, ^25^] In addition to single‐molecule layers, polymer architectures – such as linear homopolymers,^[26]^ block copolymers,^[27]^ polymer brushes,^[28]^ and crosslinked networks^[29]^ – offer interesting strategies to enhance the reactive regions of the responsive functional groups. For example, block copolymers containing responsive functional groups can form phase‐separated domains, enhancing local responsiveness and selectivity for ion transport.[^30^, ^31^, ^32^] Furthermore, densely packed polymer brushes create hierarchical structures that amplify wettability and ion‐gating changes under light stimuli.[^9^, ^33^]

While several studies have focused on the development of light‐responsive nanochannels, most works have concentrated on the structural aspects of the nanochannels and the design of photodetectors; however, fewer studies have explored the role of molecular or polymeric architectures within these confined spaces. In this study, we integrate spiropyran (Sp) ‐based polymers into anodic aluminum oxide (AAO) nanochannels to achieve tailored photoresponsive behaviors. Light irradiation induces the reversible ring‐opening isomerization of spiropyran groups, leading to changes in the packing and polarity of the polymer chains. Using solution wetting and surface‐initiated atom transfer radical polymerization (SI‐ATRP), we construct polySp‐coated and polySp‐grafted architectures. These systems exhibit unique macroscopic and microscopic responses, including reversible color changes, wettability adjustments, and modulation of ion transport under UV and visible light. The findings of this study open the door to the design of next‐generation photoswitchable nanochannels. By leveraging molecular customization, we enhance multifunctionality that can be applied in fields such as optical information storage, photogated materials, and sensors.

## Experimental Section

### Materials

2,3,3‐Trimethyl‐3H‐indole, methacryloyl chloride, and 2‐hydroxy‐5‐nitrobenzaldehyde were sourced from Sigma‐Aldrich and used as received. (3‐Aminopropyl)triethoxysilane (98 %) was bought from Alfa Aesar. 2‐Bromoethanol was supplied by Tokyo Chemical Industry.The initiator, 2‐bromoisobutyryl bromide (C_4_H_6_Br_2_O, 98 %), and the catalyst copper (I) bromide (CuBr, 98 %) were obtained from Sigma‐Aldrich. 35 wt % Hydrogen peroxide was supplied by Honeywell, and potassium chloride (KCl, 99 %) was sourced from Union Chemical. *N, N, N’, N’’, N’’*‐pentamethyldiethylenetriamine (PMDETA, 98 %) was acquired from Nova Materials. *n*‐Hexane (99.5 %) and dry tetrahydrofuran were purchased from Fisher Scientific, and acetone (99.5 %) was obtained from Echo. AAO membranes with diameters of ~13 mm and thicknesses of ~60 μm were purchased from Whatman. The detailed synthetic procedures of the spiropyran molecules and polymers are listed in the Supporting Information (Figures S1–S9).[^9^, ^34^, ^35^, ^36^]

#### Solution Wetting of Sp Molecules and Polymers in AAO Nanochannels

AAO membranes were washed with deionized water and ethanol and dried in a vacuum before use. SpOH solution in methanol (2 mg/mL) and Sp copolymer solution in chloroform (4 mg/mL) were also prepared. To coat the Sp copolymer into the AAO nanochannels, we used chloroform as a solvent rather than an aqueous solution based on their solubility. For the drop‐coating method, the AAO membranes were immersed in the Sp solutions, followed by a drying process in a vacuum for 24 h.

#### Polymer Grafting of Sp Molecules and Polymers in AAO Nanochannels

AAO membranes were first treated with an H_2_O_2_ solution, followed by immobilization of aminopropyl‐triethoxylsilane (APTES) and 2‐bromobutyryl bromide (ATRP initiators). The polySp‐grafted AAO membranes were then synthesized via SI‐ATRP, using 60 mg of SPMA monomer, 2.9 mg of CuBr, and the ATRP initiator‐grafted AAO membrane under a nitrogen atmosphere for 1 h. After the polymerization process, the polySp‐grafted AAO membranes were thoroughly washed with ethanol and dried under vacuum before further use.

#### Fabrication of the Photoswitchable PolySp‐Based Electrolyte System

A polySp‐based AAO membrane was sandwiched between two torus‐shaped silicon spacers, each covered with ITO glass. Aqueous electrolytes (KCl solution, 0.02 mg/mL) were infused into the nanopores on both sides by filling the middle hole of the spacers. For the EIS testing, the applied voltage amplitude was set to 1 V, with a frequency range of 1 Hz to 1 MHz. The fitting analyses were conducted by electrochemical fitting software (ZView2).

#### Structure Characterizations and Analyses

A Fourier‐transform infrared (FT‐IR) spectrometer (PerkinElmer Spectrum One) was used to confirm the chemical structures of the samples. To characterize the Sp molecules and polymers, an ^1^H nuclear magnetic resonance (NMR) spectrometer (JOEL 400 MHz) was applied. Ultraviolet‐visible (UV‐vis) absorbance and reflectance spectra were recorded from 350–700 nm using a Hitachi U‐4100 spectrometer. A scanning electron microscope (SEM, JEOL JSM‐7401F) was employed to investigate the surface morphologies of the samples using an acceleration voltage of 5 kV. Prior to SEM analyses, the AAO membranes were coated with a 4 nm layer of platinum. Water contact angles (WCA) were measured to assess the hydrophobicity of the samples using a goniometer (FTA 125, First Ten Ångstroms). Droplets of water (4 μL) were applied to the surfaces of the samples under ambient conditions for the measurements. A TGA 55 (TA Instruments) was applied to conduct thermal stability analyses at a temperature range of 100–650 °C under N_2_ atmosphere (heating rate: 10 °C/min). Elemental compositions were confirmed with a high‐resolution X‐ray photoelectron spectrometer (HRXPS) (PHI Quantera II) and an energy dispersive spectroscope (EDS, Oxford EDS 7585). Resistance changes were measured with electrochemical impedance spectroscopy (EIS) measurements (CHI6000, CHI instruments) over a frequency of 1 kHz and a voltage of 1 V with and without UV irradiation.

## Results and Discussion

**Figure 1:**
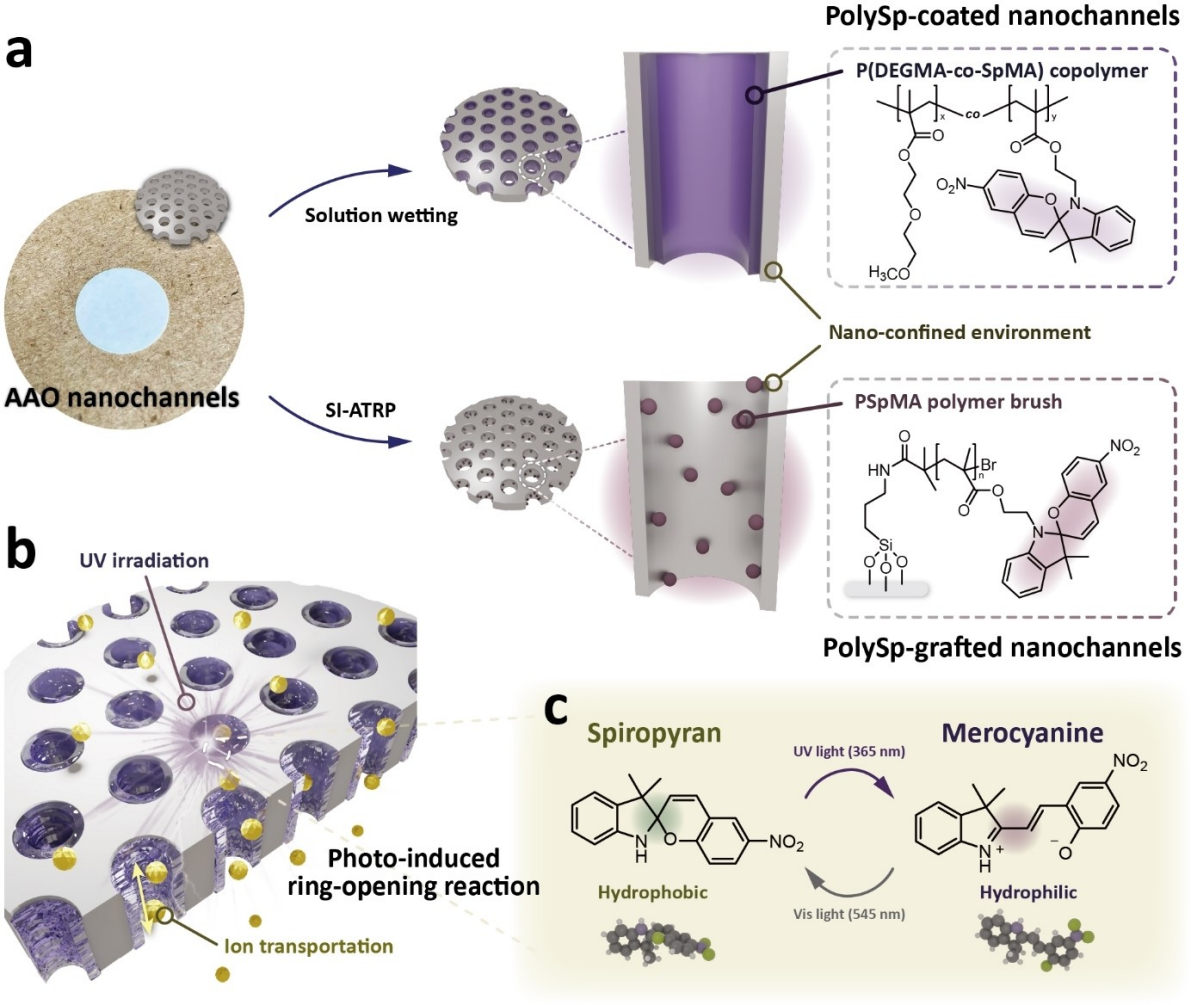
Conceptual illustration of the polySp‐based nanochannels. (a) Graphical illustration, photograph, and chemical structures of pure AAO nanochannels and polySp‐coated and polySp‐grafted AAO nanochannels. (b) Schematic illustration of the photoswitable SP polymer electrolyte system. (c) Photoisomerization of SP and related hydrophobicity changes via UV and visible lights.

Figure 1 illustrates the conceptual design of the polySp‐based nanochannels. The nanoconfined environment is constructed using commercial AAO templates with pore diameters of ~200 nm, channel lengths of ~60 μm, and open pore structures on both sides. Figure 1a presents a photograph and schematic of the AAO templates. Serving as both containers and reactors for spiropyran‐based polymers, the AAO membranes undergo solution wetting and surface‐initiated atom transfer radical polymerization (SI‐ATRP) for coating and grafting, respectively. In the polySp‐coated AAO system, spiropyran copolymers, P(DEGMA‐co‐SpMA), are introduced into the nanochannels via solution wetting, creating a polymer matrix. Conversely, in the polySp‐grafted AAO system, spiropyran homopolymers, PSpMA, are covalently linked to the channels through SI‐ATRP, forming polymer brush‐like structures. Following these modifications, photoswitchable composite polymer electrolytes are fabricated by infiltrating aqueous electrolyte solutions into the polySp‐based AAO membranes, as depicted in Figure 1b.

By shining UV light, spiropyran molecules can undergo UV‐triggered ring‐opening reactions and convert into zwitterionic merocyanine structures, exhibiting notable macroscopic (e. g., color) and microscopic (e. g., polarity) changes (Figure 1c).^[19]^ Initially, the spiropyran functional groups in the polySp‐based nanochannels are hydrophobic because of their ring‐closed polymeric nature. Under UV irradiation, however, heterolytic cleavage of the C−O bond increases the dipole moment from ∼4.3 to ∼17.7 D, rendering the surfaces more hydrophilic.^[37]^ This shift alters the ionic transport environment, enabling reversible conditions favoring water inclusion or exclusion and allowing ion mobility enhancement or suppression. Both polySp‐coated and polySp‐grafted AAO membranes exhibit comparable tendencies but differing extents of photochromism and light‐induced ionic control.

Most research on light‐responsive nanochannels has focused on functionalized single nanochannels, with molecule‐modified channels preferred due to their easier synthesis. In this work, we compare the coating and grafting of single spiropyran molecules and polymers, both of which enhance the reactive area and responsiveness of the systems. We hope that the molecule/polymer architectures discussed in this work will lead to the exploration of better designs or structures in diverse applications, including sensors,^[20]^ light‐gating materials, and information storage.^[9]^

**Figure 2:**
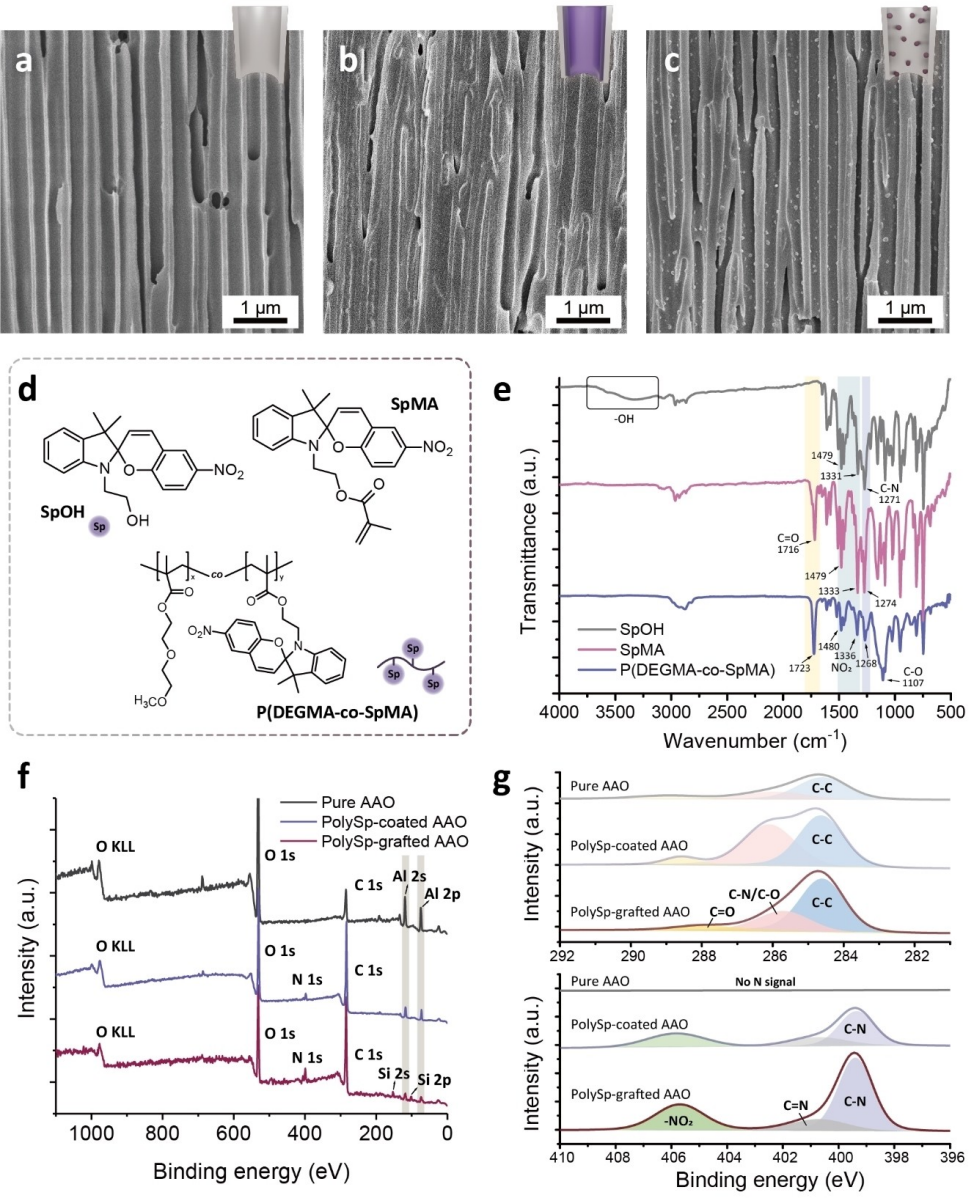
Structural characterizations of the polySp‐based AAO nanochannels. (a–c) Side‐view SEM images of (a) pure AAO and (b) polySp‐coated and (c) polySp‐grafted nanochannels. (d) Chemical structures of the SP molecules and polymer. (e) FTIR spectra of SP molecules and polymer. (f) Overall XPS survey spectra and (g) high‐resolution XPS spectra centered on C 1s and N 1s scans of pure AAO and polySp‐coated and polySp‐grafted nanochannels.

Figure 2a presents side‐view scanning electron microscope (SEM) images of pure AAO, revealing an average pore size of ~230 nm and a porosity of ∼40 %. After polySp‐based modifications, the AAO nanochannels exhibit distinct nanopore morphologies, as shown in Figure 2b and c. In the polySp‐coated AAO membranes (Figure 2b), most of the AAO surfaces are covered with P(DEGMA‐co‐SpMA) copolymers. Surface tension differences, capillary action, and hydrophilic effects or hydrogen bonding all influence the polymer distribution within the nanochannels, resulting in a complete wetting regime in the polySp‐coated AAO system. In contrast, the polySp‐grafted AAO membranes (Figure 2c) display dispersed PSpMA polymer clusters randomly distributed along the sidewalls of the nanochannels.

The chemical structures of the spiropyran molecules or monomers (e. g., SpOH and SpMA) and the P(DEGMA‐co‐SpMA) copolymer are shown in Figure 2d. Distinct functional groups of these derivatives enable the versatile decoration of AAO nanochannels with spiropyran molecules and polymers using wetting methods or chemical reactions, which can also be validated through Fourier‐transform infrared (FTIR) spectroscopy (Figure 2e). SpOH, SpMA, and the P(DEGMA‐co‐SpMA) exhibit structural similarities based on the presence of spiropyran groups, including NO_2_ stretching vibrations and C−N stretching of the amine group. Compared with the monomers, P(DEGMA‐co‐SpMA) exhibits a peak at 1107 cm^−1^, corresponding to the C−O stretching of the ether groups in DEGMA. Additionally, peaks at 1716 and 1723 cm^−1^, associated with C=O stretching, are observed in SpMA and P(DEGMA‐co‐SpMA) but are absent in SpOH. The broad peaks from 3000–3700 cm^−1^ in SpOH arise from O−H stretching vibrations.

X‐ray photoelectron spectroscopy (XPS) further verifies the successful coating and grafting of spiropyran polymers. The overall survey spectra of the samples (Figure 2f) show peaks at 74 and 118 eV, corresponding to Al 2p and Al 2 s of aluminum oxide, respectively. In the C 1s scan (Figure 2g), the C=O peaks shift from 289.1 eV in pure AAO to 288.5 eV in polySp‐coated AAO and 287.94 eV in polySp‐grafted AAO, reflecting increased electron density in the polymer structures. In the N 1s scan, peaks corresponding to the nitro group (~405.7 eV) and pyrroline (~400.7 eV) are evident in both polySp‐modified AAOs, while no signal is detected in pure AAO membranes. TGA and EDS analyses are used to characterize the amount and the elements of the Sp polymer in polySp‐grafted AAO, as presented in Figure S10 and Table S1.

**Figure 3:**
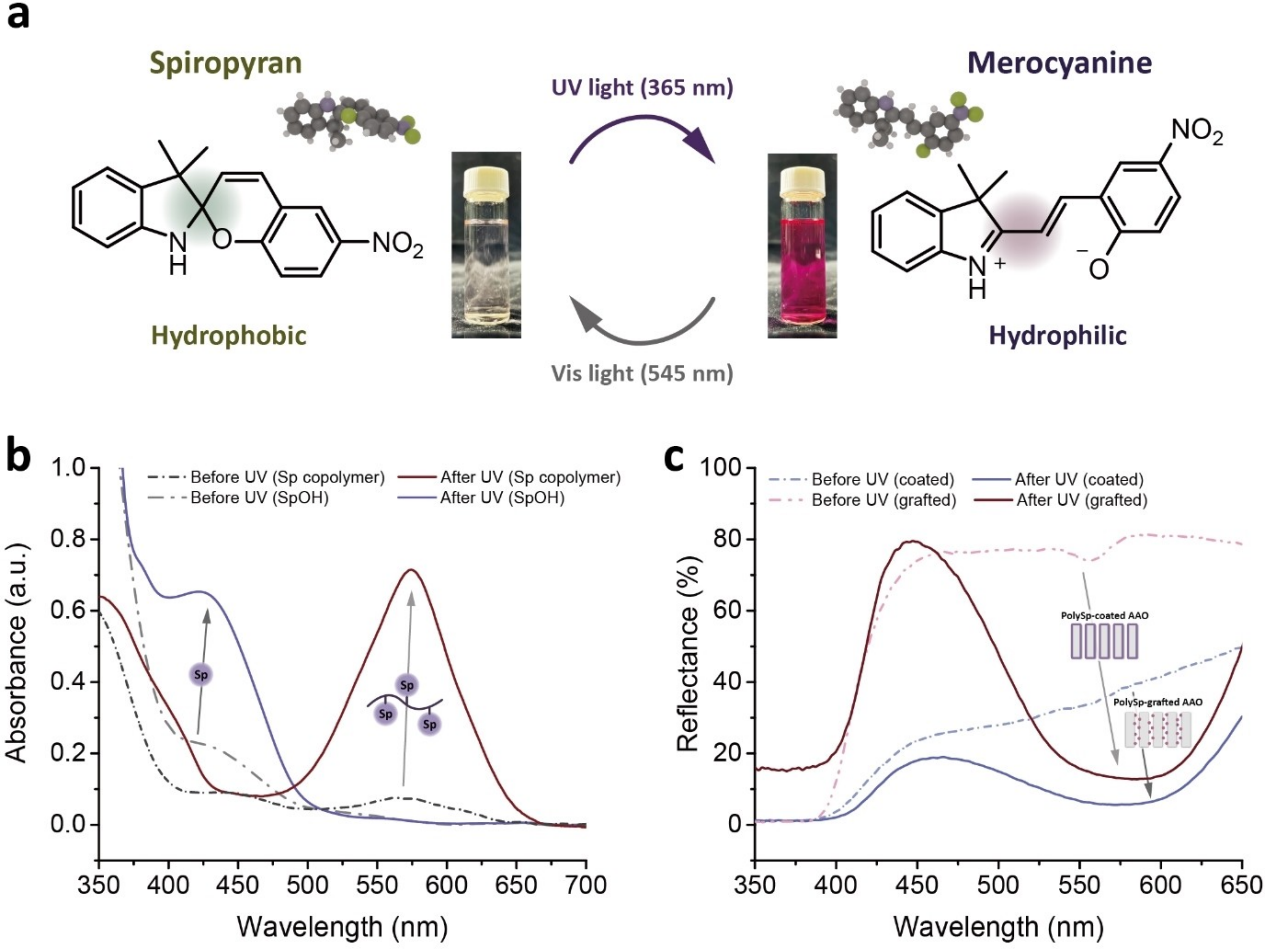
Photoswitching and the ring‐opening reaction of the spiropyran and merocyanine solutions and polySp‐based AAO membranes. (a) Reaction mechanism and photographs of the solutions before and after UV irradiations. (b) UV–vis absorption spectra of the SpOH and Sp copolymer solutions before and after UV irradiations. The SpOH and Sp copolymer are dissolved in methanol and chloroform, respectively, due to their differing solubility. (c) UV–vis reflectance spectra of the polySp‐coated and polySp‐grafted AAO nanochannels before and after UV irradiations.

The photoswitchable properties of spiropyran molecules under various light irradiations have been widely reported.[^19^, ^37^] Figure 3a illustrates the chemical structures and solution colors of spiropyran and merocyanine. Upon UV light exposure, the heterolytic cleavage of the spiropyran's C−O bond occurs, resulting in the ring‐opening formation of merocyanine, which exhibits a bright purple color. This reaction is reversible under visible light irradiation, restoring the closed‐ring, nonpolar spirocyclic form of spiropyran, which appears as a colorless solution. The color changes during photoisomerization can be monitored using UV–vis absorption spectroscopy, as shown in Figure 3b. Within 3 s of UV exposure, the closed spiropyran molecules absorb photons, initiating a photochemical reaction that breaks the spiro C−O bond and opens the spiro‐ring. This process produces new absorption peaks at ~425 nm for SpOH and ~574 nm for P(DEGMA‐co‐SpMA), corresponding to the gradual formation of merocyanine‐like open‐ring structures, which is responsible for the observed coloration. As reported in previous studies, the side chain effect in spiropyran significantly influences its electronic environment, modifying the absorption wavelength by affecting the stability of the merocyanine state.^[38]^ Likewise, the solvent effect plays a pivotal role, with variations in polarity and solvation dynamics shifting the equilibrium between spiropyran and merocyanine forms, resulting in distinct UV–vis absorption spectra.[^39^, ^40^] Figure 3c demonstrates the photochromism of the polySp‐based AAO membranes in the solid state, analyzed via UV–vis reflectance spectroscopy. Upon UV exposure, the spiropyran groups in the polymer nanochannels transition to their merocyanine states within seconds, reducing reflectance at 581 and 589 nm for polySp‐coated and polySp‐grafted AAO, respectively. These changes are also visually detectable to the naked eye.

**Figure 4:**
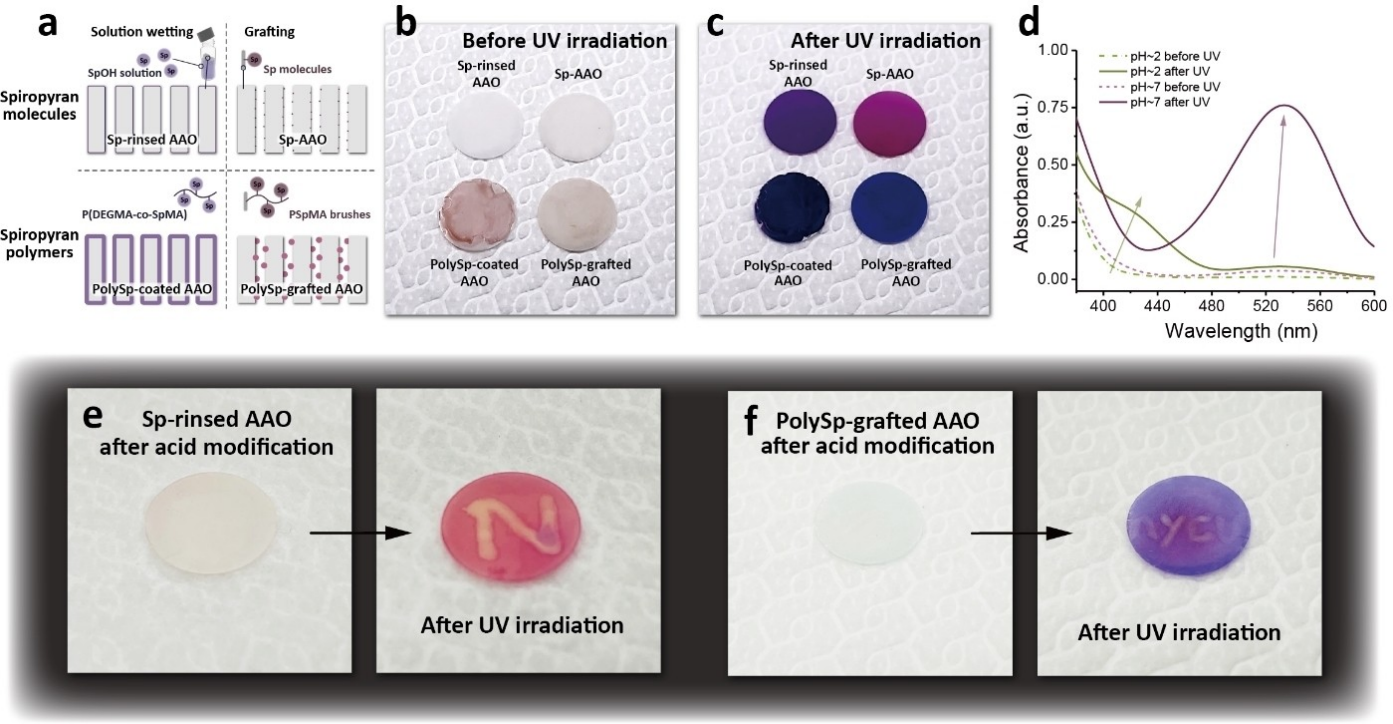
Photoswitchable color changes of the polySp‐based nanochannels. (a) Schematic illustration of different polySp‐modified AAO nanochannels via molecules and polymers solution wetting and grafting. (b,c) Photographs of Sp‐based nanochannels (b) before and (c) after UV irradiations. (d) UV–vis absorption spectra of the SpOH solutions in different pH values. (e, f) Photographs of Sp‐based nanochannels after acid modifications: (e) Sp‐rinsed and (f) polySp‐grafted AAO nanochannels before and after UV irradiations.

The photochromism of polySp‐based AAO membranes offers an exciting potential for imaging and detection, as illustrated in Figure 4. Figure 4a depicts the various spiropyran modifications of AAO membranes, including spiropyran molecules and polymers introduced through solution‐wetting and grafting processes onto the nanochannels. The corresponding appearances of the Sp‐based AAO membranes are shown in Figure 4b. Compared with membranes modified with spiropyran molecules, polySp‐based AAO membranes exhibit pink surfaces because of the thicker polymer layers, which may also hinder the structural transitions between isomers. Upon UV irradiation for 3 s, the Sp‐based AAO membranes develop varying shades of purple, indicating the merocyanine states of the functional groups within the AAO and polymer structures. Compared to Sp‐AAO and Sp‐rinsed AAO, polySp‐based AAO membranes exhibit darker purple surfaces due to the higher density of spiropyran groups in the polymer side chains, as shown in Figure S11. During the ring‐closing reaction under visible light irradiation, differences between the molecular and polymer architectures become apparent, resulting in variations in reaction rates. For AAOs modified with spiropyran molecules, the color change occurs within 30 seconds. In contrast, polySp‐based AAOs require at least 5 minutes to transition from purple back to white (Figure S12).

In addition to light‐induced color changes, spiropyran demonstrates chromism in response to pH variations. Figure 4d shows the UV–vis absorption spectra, highlighting the interconversion of spiropyran groups under different pH conditions. At acidic condition (pH ~2), the ring‐opened merocyanine undergoes protonation, eliminating the absorbance at 535 nm observed under neutral conditions (pH ~7) and resulting in a colorless solution. To further explore the photochromism of Sp‐based AAO membranes under acidic and basic conditions, Sp‐rinsed and polySp‐grafted AAOs are selected for their patternable properties, as shown in Figures 4e and f, respectively. To create patterns on the AAO membranes, regions are selectively treated with 0.1 M HCl_(aq)_, which become invisible after drying. Upon UV exposure, hidden patterns emerge owing to the distinct configurations of purple merocyanine and colorless protonated merocyanine states. This phenomenon demonstrates potential applications in photo‐induced information encryption.

**Figure 5:**
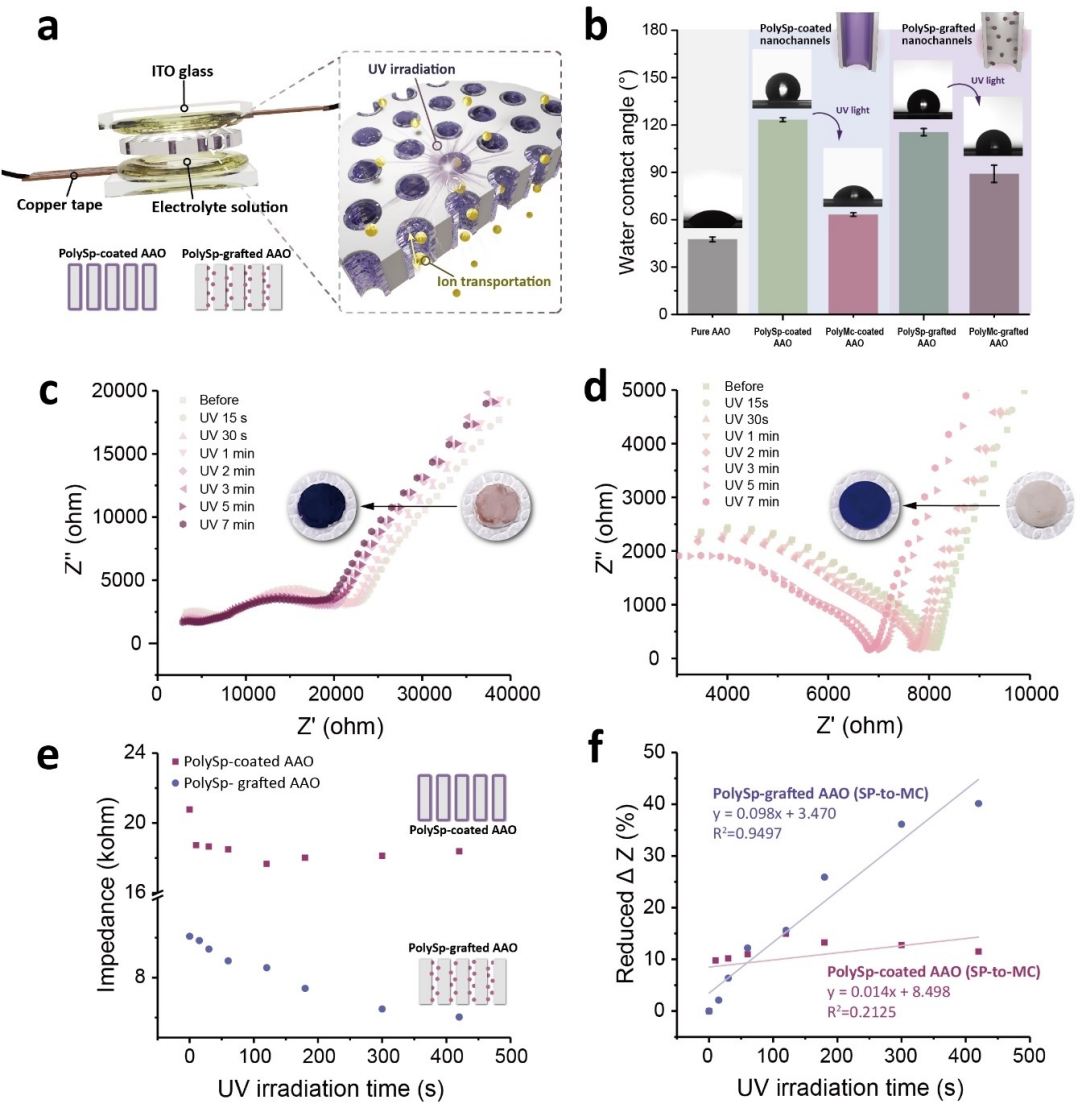
Photoswitching of the electrochemical properties in the polySP‐based AAO nanochannels. (a) Schematic illustration of the electrode configuration and mechanism. (b) Water contact angle plots and images of the pure AAO and polySp‐coated and polySp‐grafted nanochannels before and after UV irradiations. The measurement is performed five times on the same sample, with the error bars representing the standard deviation. (c, d) Electrochemical impedance spectra of the (c) polySp‐coated and (d) polySp‐grafted nanochannels upon UV irradiations. (e) Plots of impedance and (f) impedance changes of the polySp‐coated and polySp‐grafted nanochannels with different UV irradiation times.

In addition to macroscopic color changes, the photoinduced wettability and ion‐gating behaviors of polySp‐coated and polySp‐grafted AAO membranes are investigated, as shown in Figure 5. Impedance changes are recorded under light stimulation by applying a voltage of 1 V, as illustrated in Figure 5a. Aqueous electrolyte solutions (KCl, 0.02 mg/mL) are confined in containers made of ITO glasses and Teflon spacers with the polySp‐based AAO membranes sandwiched between two copper electrodes with a working area of 0.92 cm^2^, facilitating ion transport through the nanochannels. To assess the stability of the polySp‐coated AAO system in aqueous electrolyte solutions, the samples are washed three times with water. The results confirm that the Sp copolymer layers are difficult to remove with nonsolvents such as water (Figure S13 and Table S2). As a result, the non‐covalent bond connection does not cause instability in the polymer connection or affect the functionality of the photoresponsive ion nanochannel when used with aqueous electrolyte (KCl) solutions. If chloroform or another good solvent for the Sp copolymer is used, however, the polymer could be removed due to the non‐bonding nature of its adhesion to the nanochannel walls.

Figure 5b highlights the photocontrollable wettability of the polySp‐based AAO membranes, a critical factor influencing the impedance changes upon UV light exposure. Initially, pure AAO membranes exhibit hydrophilic properties with a water contact angle (WCA) of 47.6°±1.5°, attributed to the hydroxyl groups on the AAO surface. After modification via solution wetting and grafting processes, the WCA increases to 123.4°±1.3° and 115.5°±2.27° for polySp‐coated and polySp‐grafted AAOs, respectively, reflecting the nonpolar nature of spiropyran groups and the alkyl chains in the polymer structures. Upon UV irradiation for 10 s, the WCA decreases to 63.3°±1.1° and 89.1°±5.5° for polySp‐coated and polySp‐grafted AAOs, respectively, due to the polar ring‐opened merocyanine groups and charge redistribution. Notably, the higher hydrophobicity of the polySp‐grafted AAO arises from the hierarchical structures of the grafted polymer within the AAO channels.

The photocontrollable wettability directly influences ion conductivity in both polySp‐coated and polySp‐grafted AAO membranes, as shown in Figure 5c and d. Electrochemical impedance spectroscopy (EIS) is employed to monitor the reduction in impedance over time upon UV exposure. The overall impedance magnitude (Z) for both systems gradually decreases with increasing UV irradiation duration, as reflected in the trends shown in Figure 5e. Figure 5f summarizes the photoinduced ion gating behaviors for both polySp‐coated and polySp‐grafted AAO membranes.

Interestingly, while both systems exhibit impedance changes over extended durations (up to 7 min), their behaviors differ because of the variations in polymer structures. In the polySp‐coated AAO system, impedance drops rapidly with a ΔZ reduction of 9.8 % within the first 15 s, stabilizing at ~11.5 % after 7 min of UV irradiation. This result indicates that ion gating occurs predominantly in the initial seconds, with little change thereafter. Conversely, the polySp‐grafted AAO system shows a more gradual impedance reduction with a ΔZ decrease of 2.1 % initially, eventually reaching ~40.2 % after 7 min, suggesting a broader window for ion controllability over time. These distinct photoresponsive behaviors of the polySp architectures highlight the potential to tailor polymer structures within nanochannels for diverse applications, including light‐gating materials, sensors, and information storage.

## Conclusions

In this work, we demonstrate the design and development of photoresponsive nanochannels by incorporating spiropyran‐based polymers into AAO templates. The integration of these spiropyran functional groups enables reversible photoisomerization under UV and visible lights, which triggers significant macroscopic and microscopic changes in the nanochannels. These changes include color transitions, shifts in wettability, and modulation of ion transport, offering promising potential for dynamic and controlled applications in sensors, photogated materials, and optical information storage. The comparison between the polySp‐coated and polySp‐grafted AAO systems reveals that both architectures exhibit light‐induced ionic control, though with varying extents of photochromic behavior. These findings underscore the importance of molecular design in optimizing the functionality of photoresponsive nanochannels. Additionally, the study highlights the versatility of spiropyran‐based nanochannels for various applications, including information encryption and enhanced sensing capabilities.

## Conflict of Interests

The authors declare no conflict of interest.

1

## Supporting information

As a service to our authors and readers, this journal provides supporting information supplied by the authors. Such materials are peer reviewed and may be re‐organized for online delivery, but are not copy‐edited or typeset. Technical support issues arising from supporting information (other than missing files) should be addressed to the authors.

Supporting Information

## Data Availability

The data that support the findings of this study are available from the corresponding author upon reasonable request.
