# Supplementary material for: Tailoring Polymer Coatings and Grafting Structures for Photoswitchable Ionic Transport in Solid‐State Nanochannels
Source: Chem Asian J. 2025 Mar 12;20(7):e202401684. doi: 10.1002/asia.202401684 (PMC11980769; doi:10.1002/asia.202401684)
Supplement: Supplementary file 1 — Supporting Information [file ASIA-20-e202401684-s001.pdf]

Chemistry - An Asian Journal

Supporting Information

Tailoring Polymer Coatings and Grafting Structures for Photoswitchable Ionic Transport in Solid-State Nanochannels

Yi-Fan Chen, Vaishali Pruthi, Yu-Chun Liu, Cheng-Yeh Yang, Lin-Ruei Lee, Ming-Hsuan Chang, Chun-Chi Chang, Patrick Théato,* and Jiun-Tai Chen*

Supporting Information

Tailoring Polymer Coatings and Grafting Structures for Photoswitchable Ionic Transport in Solid-State Nanochannels

Yi-Fan Chen,[1] Vaishali Pruthi,[2] Yu-Chun Liu,[1] Cheng-Yeh Yang,[1] Lin-Ruei Lee,[1] Ming-Hsuan Chang,[1] Chun-Chi Chang,[1] Patrick Théato,[2,3]* and Jiun-Tai Chen[1,4]*

[1]Department of Applied Chemistry, National Yang Ming Chiao Tung University, 300093 Hsinchu, Taiwan

[2]Institute for Chemical Technology and Polymer Chemistry (ITCP), Karlsruhe Institute of Technology (KIT), Kaiserstraße 12, D-76131 Karlsruhe, Germany

[3]Soft Matter Synthesis Laboratory Institute for Biological Interfaces III, Karlsruhe Institute of Technology (KIT), Hermann-von-Helmholtz-Platz 1, D-76344 Eggenstein-Leopoldshafen, Germany

[4]Center for Emergent Functional Matter Science, National Yang Ming Chiao Tung University, 300093 Hsinchu, Taiwan

Synthesis of Spiropyran Molecule 1 (SpOH)

SpOH was synthesized with modifications by previously reported methods,[1] as outlined in Figure S1. 2,3,3-Trimethyl-3H-indole (2.6 g, 16 mmol) and 2-bromoethanol (2.46 g, 20 mmol) were mixed in 20 mL of acetonitrile and refluxed at 85 °C under N_2 for 24 h. After cooling to room temperature, the solvent was removed via rotary evaporation. The residue was suspended in 30 mL of hexane, sonicated, and filtered. The resulting intermediate bromide salt was crystallized using chloroform.

The bromide salt (2.93 g, 10 mmol) was dissolved in 50 mL of 0.3 M $KOH_{(aq)}$ and stirred at room temperature for 15 min. The solution was extracted with diethyl ether, and the organic phase was concentrated to yield the oxazole derivative.

The oxazole derivative (9,9,9a-trimethyl-2,3,9,9a-tetrahydro-oxazolo[3,2-a]indole, 1.09 g, 5 mmol) and 2-hydroxy-5-nitrobenzaldehyde (1.25 g, 7.5 mmol) were dissolved in 10 mL of ethanol and refluxed at 65 °C under N_2 for 24 h. After cooling to room temperature, the solution was concentrated via rotary evaporation, yielding a dark purple solid.

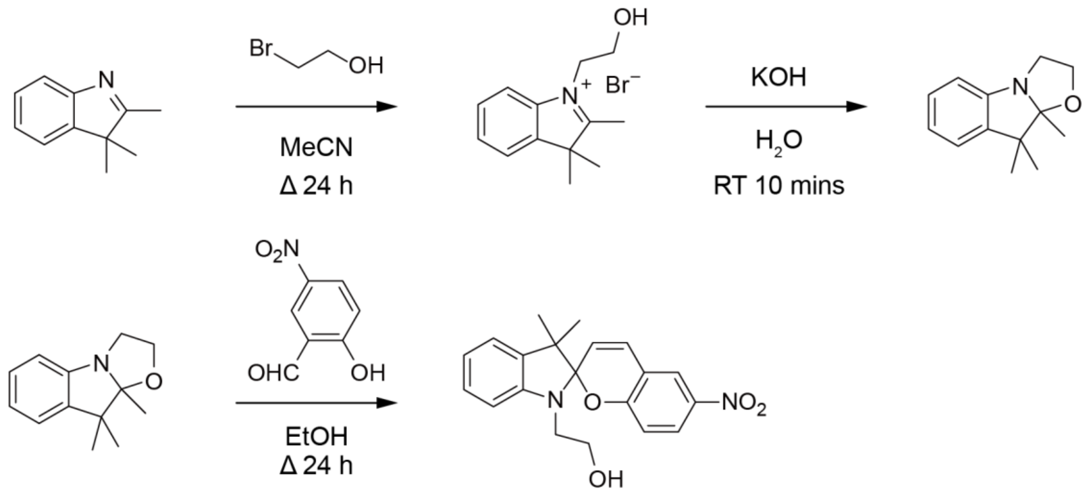

Figure S1. Synthetic scheme of the spiropyran molecule (SpOH).

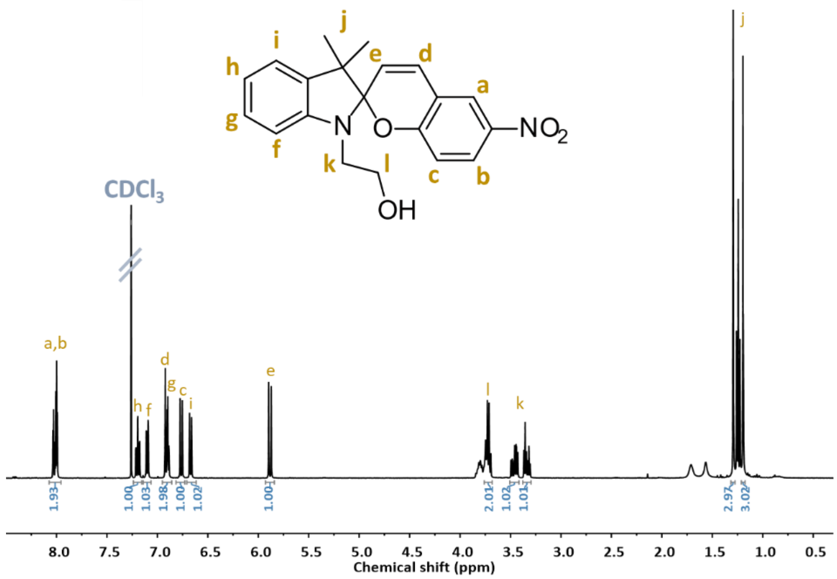

Figure S2. ^{1}H NMR spectrum of the spiropyran molecule (SpOH).

Synthesis of Spiropyran Monomer (SpMA)

SpOH (1.05 g, 3 mmol), methacryloyl chloride (0.784 g, 7.5 mmol), Et_3N (0.758 g, 7.5 mmol), and CH_2Cl_2 (40 mL) were added to a round-bottom flask at 0 °C. The mixture was stirred for 10 h and then filtered and extracted with a $NaHCO_3$/water solution (1:1).[1, 2] The organic layer was dried with $MgSO_4$ and concentrated under reduced pressure to yield a crude purple solid. The crude product was purified via column chromatography using an ethyl acetate/hexane (1:1) eluent. The purified compound was crystallized from 95% ethanol several times to obtain SpMA as a pure product.

In the chemical shift range of 1.25 to 2.0 ppm, the impurities can be attributed to residual solvents used during purification, including ethyl acetate, hexane, and ethanol. These impurities could be further eliminated through vacuum drying and N_2 purging in the grafting process of polySp-grafted AAO. During the subsequent polymerization process, the impurities (mainly residual solvents) should have already been removed and have a minimum effect on the reactions. In the chemical shift range of 5.5 to 7.5 ppm, some small peaks corresponding to the merocyanine states of the compound can be seen, which are commonly observed in the NMR spectra of spiropyran-based molecules.

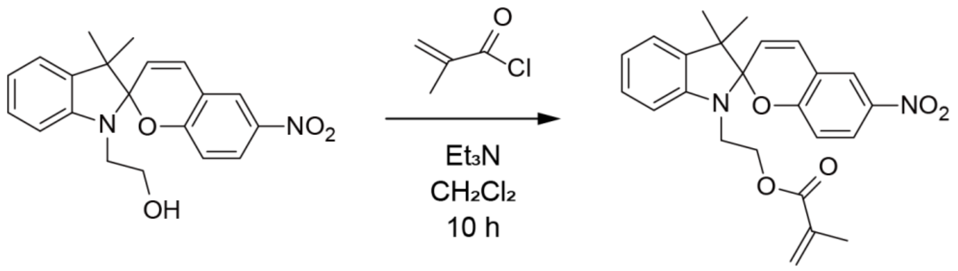

Figure S3. Synthetic scheme of the spiropyran monomer (SpMA).

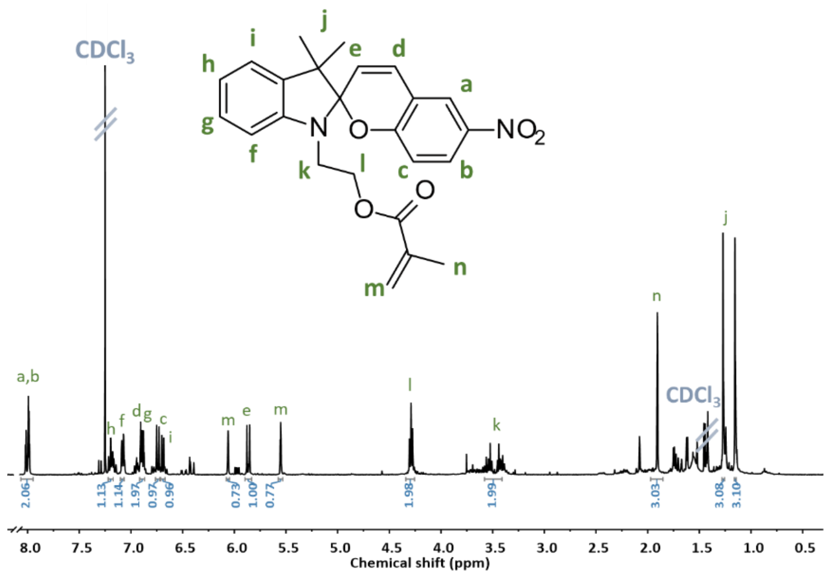

Figure S4. ^{1}H NMR spectrum of the spiropyran monomer (SpMA).

Grafting Process of the PolySp-grafted AAO

To increase surface OH group density, pristine AAO membranes were immersed in 34 wt % H_2O_2 solution for 30 min and then rinsed thoroughly with deionized water and ethanol. Following H_2O_2 pretreatment, the membranes were immersed in 5% aminopropyltriethoxysilane (APTES) solution for 30 min to achieve silanization, forming APTES-grafted AAO membranes with surface-expressed NH_2 groups. The membranes were then rinsed with ethanol and dried under reduced pressure.

The APTES-grafted membranes were placed in a 25 mL round-bottom flask with 4 mL triethylamine and 10 mL dichloromethane. After N_2 purging for 15 min in an ice-water bath, 2-bromobutyryl bromide was added to create an ATRP surface initiator. The reaction was maintained in the ice bath for 1 h and then continued at room temperature for 12 h. The membranes were washed with dichloromethane and vacuum-dried.

For surface-initiated atom transfer radical polymerization (SI-ATRP), SPMA (60 mg), CuBr (2.9 mg), and ATRP initiator-grafted AAO membranes were placed in a 25 mL round-bottom flask. Dry THF (6 mL) and ethyl α-bromoisobutyrate (EBiB, 1.5 μL) were added under an N_2 atmosphere and stirred for 1 h. Subsequently, PMDETA (12.8 μL) was quickly injected, and the reaction was carried out at 60 °C in an oil bath for 24 h. After SI-ATRP, the polySp-grafted AAO membranes were washed with ethanol and dried under vacuum.

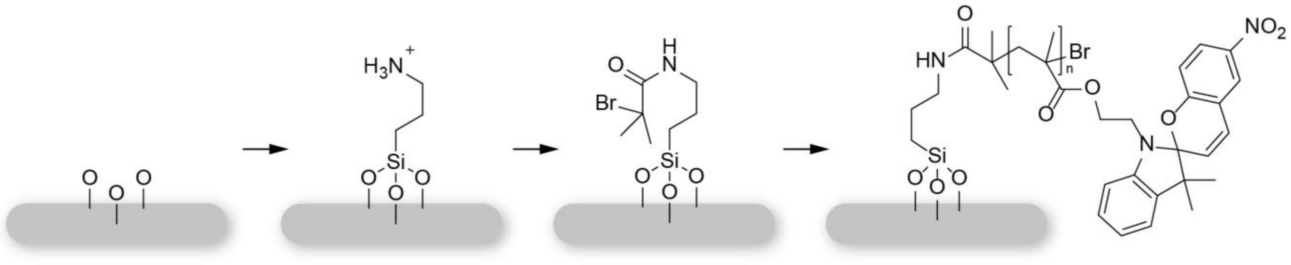

Figure S5. Synthetic scheme of the grafting process of the polySp-grafted AAO.

Synthesis of Spiropyran Molecule 2 (SpCOOH)

The spiropyran molecule 2 SpCOOH was synthesized using a modified procedure based on previous reports. Initially, a mixture of 2,3,3-trimethylindolenine (4.0 g, 25 mmol) and 3-iodopropionic acid (5.4 g, 27 mmol) in toluene (10 mL) was refluxed under a nitrogen atmosphere for 12 h with stirring. After cooling to room temperature, the reaction mixture was filtered, and the resulting precipitate was thoroughly washed with cold hexane and diethyl ether before being vacuum-dried to yield an iodide salt intermediate.

Subsequently, the iodide salt (4.5 g, 12.6 mmol), 5-nitrosalicylaldehyde (2.1 g, 12.6 mmol), and piperidine (1.3 mL, 13.2 mmol) were combined in a round-bottom flask and refluxed for 5 h. The mixture was then stirred at room temperature for an additional 5 h. Upon completion of the reaction, the mixture was cooled to 0 °C, and the light green precipitate (SpCOOH) was collected by filtration and washed repeatedly with cold ethanol.

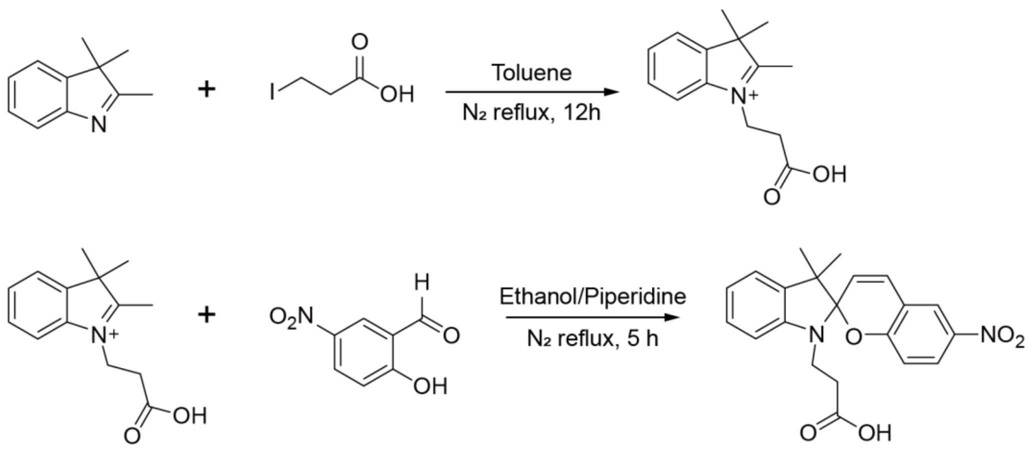

Figure S6. Synthetic scheme of the spiropyran molecule (SpCOOH).

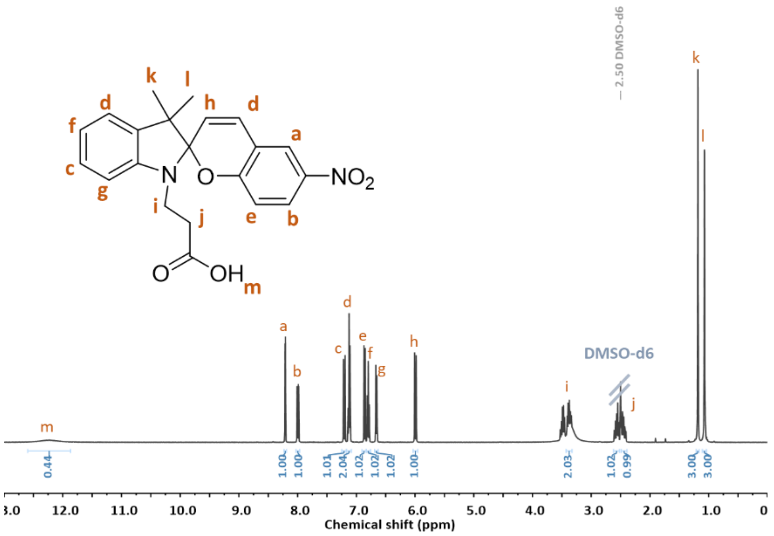

Figure S7. ^{1}H NMR spectrum of the spiropyran molecule (SpCOOH).

Grafting Process of the Sp-grafted AAO

AAO membranes were immersed in 35 wt% hydrogen peroxide at 30 °C for 1 h.[3] After treatment, the membranes were removed, rinsed, and vacuum-dried. A 20 vol % APTES solution in toluene was prepared. The H_2O_2-treated AAO membranes were immersed in the solution at 30 °C for 12 h. The membranes were then washed with toluene and acetone and vacuum-dried. SpCOOH (0.038 g) was dissolved in 10 mL ethanol, followed by 0.115 g NHS and 0.191 g EDC. The APTES-modified AAO membranes were immersed in the solution at room temperature for 24 h. Finally, the membranes were washed with ethanol and vacuum-dried.

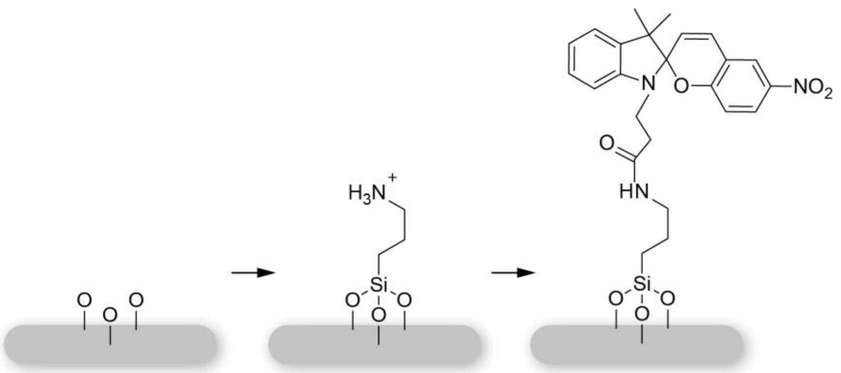

Figure S8. Synthetic scheme of the grafting process of the Sp-grafted AAO.

Synthesis of Spiropyran Copolymer (P(DEGMA-co-SpMA))

The polymerization of SpMA and DEGMA with 1 mol% AIBN as the initiator was carried out in 1,4-dioxane at 70 °C overnight. The resulting polymers were purified by precipitation in petroleum ether to remove unreacted small molecules.

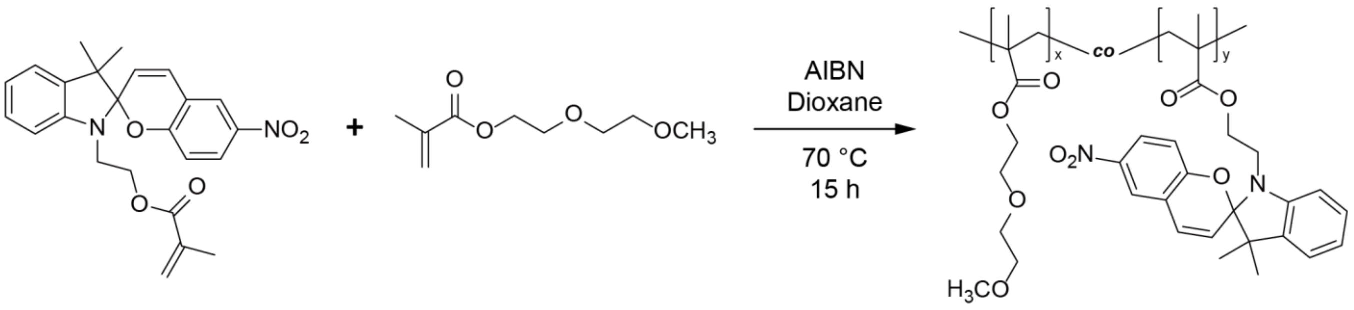

Figure S9. Synthetic scheme of the spiropyran copolymer (P(DEGMA-co-SpMA)).

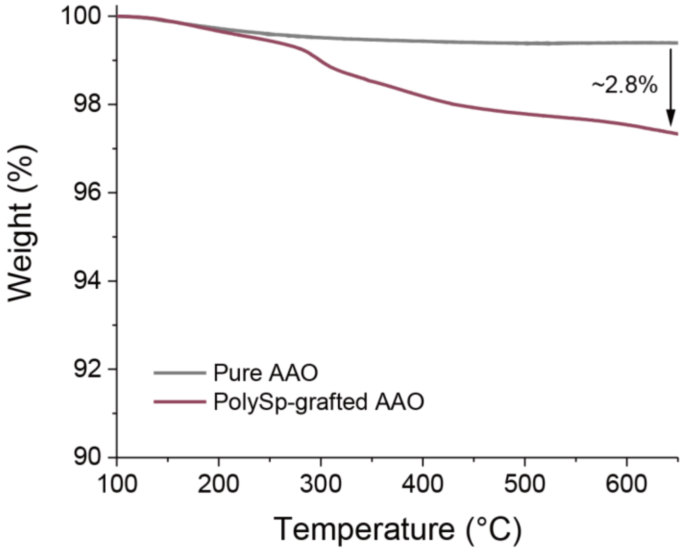

Figure S10. TGA curves of the pure AAO and the polySp-grafted AAO membrane.

Table S1. Atomic ratios of the polySp-grafted AAO membrane

**PolySp-grafted AAO**	**Atomic %**
**C**	18.68
**N**	7.22
**O**	60.23
**Al**	12.81
**Si**	0.25

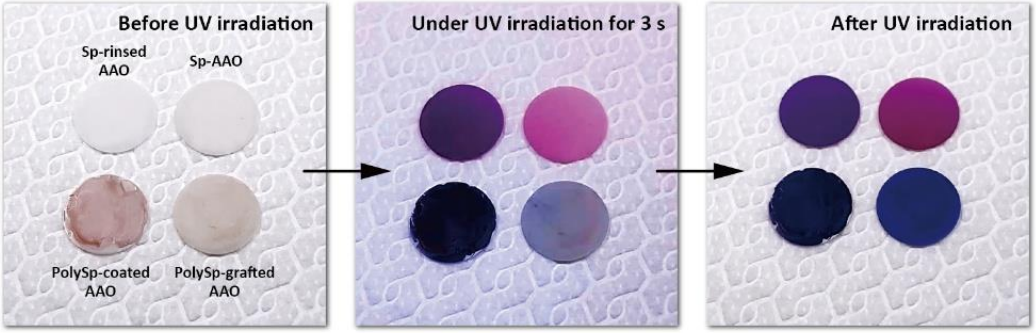

Figure S11. Photographs of Sp-based nanochannels during UV irradiations.

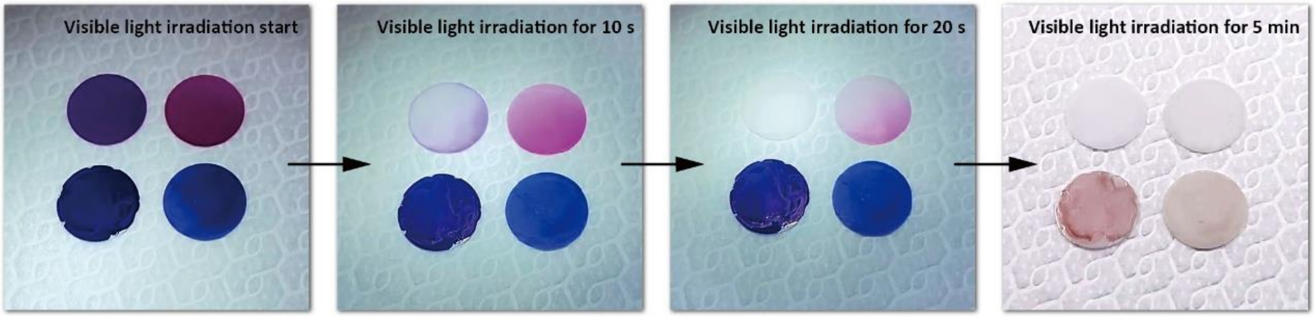

Figure S12. Photographs of Sp-based nanochannels during visible light irradiations.

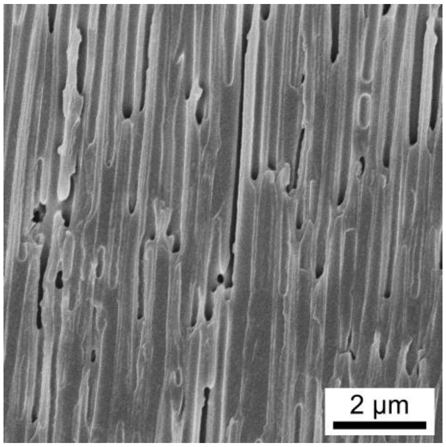

Figure S13. Side-view SEM image of the polySp-coated nanochannels after being washed by water.

Table S2. Atomic ratios of the polySp-coated AAO membranes before and after being washed by water

PolySp-coated AAO	Atomic %	
	**Before**	**After**
**C**	36.66	33.40
**N**	4.10	4.69
**O**	48.37	50.54
**Al**	10.87	11.38

References

1. Chen, Y.-F., Pruthi, V., Lee, L.-R., Liu, Y.-C., Chang, M.-H., Théato, P. and Chen, J.-T., Illuminating Biomimetic Nanochannels: Unveiling Macroscopic Anticounterfeiting and Photoswitchable Ion Conductivity via Polymer Tailoring. *ACS Nano* **2024,** *18*, 26948-26960.

2. Pruthi, V., Akae, Y. and Théato, P., Photoresponsive Spiropyran and DEGMA-Based Copolymers with Photo-Switchable Glass Transition Temperatures. *Macromol. Rapid Commun.* **2023,** *44*, 2300270.

3. Lee, L.-R., Karapala, V. K., Lin, Y.-L., He, H.-C. and Chen, J.-T., Intelligent Environmental Sensing: Fabrication of Switchable, Reusable, and Highly Sensitive Gas Sensors with Spiropyran-Grafted Anodic Aluminum Oxide Templates. *J. Phys. Chem. C* **2020,** *124*, 11870-11876.
